# Rh(III) Aqueous Speciation with Chloride as a Driver for Its Extraction by Phosphonium Based Ionic Liquids

**DOI:** 10.3390/molecules24071391

**Published:** 2019-04-09

**Authors:** Lenka Svecova, Nicolas Papaïconomou, Isabelle Billard

**Affiliations:** University Grenoble Alpes, CNRS, LEPMI, 38 000 Grenoble, France; lenka.svecova@grenoble-inp.fr (L.S.); nicolas.papaiconomou@grenoble-inp.fr or Nicolas.PAPAICONOMOU@univ-cotedazur.fr (N.P.)

**Keywords:** extraction, Rh(III), ionic liquid, speciation

## Abstract

In this work, the aqueous speciation of Rh(III) in chloride medium was investigated by UV-vis spectroscopy for ligand to metal ratios R = (Cl^−^)/(Rh) ranging from 300 to 5000, at fixed Rh concentration (2.4 × 10^−3^ M). Under the chemical conditions of this work, no time evolution was observed, which allows for the fitting of the UV-vis data by Principal Component Analysis (PCA) and Multi-Curve Resolution (MCR). From this, and by comparison with literature data, the three independent species [RhCl_4_]^−^, [RhCl_5_]^2−^ and [RhCl_6_]^3−^ were identified, their individual absorption spectra derived and their respective contribution to the collected experimental UV-vis spectra calculated. Then, extraction of Rh(III) towards the ionic liquid trihexyltetradecylphosphonium chloride was performed. Comparison with the speciation data gives insight into the extraction mechanism and the extracted species.

## 1. Introduction

In recent years, there has been a considerable renewal of interest in liquid-liquid extraction of platinum group metals (PGM), owing to their scarcity on earth and their increased use in automotive catalysts or other technological objects, that lead to no other choice than efficient recycling. Among PGM, Rh appears as the trickiest element to separate from a mixture of Pt, Pd and Ru dissolved in chloride media. With this aim, several extracting systems were investigated, such as molecular solvents combined to various extracting agents [1,2,3] or mixtures of imidazolium-based [2], ammonium-based [3] or phosphonium-based ionic liquids (ILs). For the latter family, these solvents were used either pure [4,5] or diluted in toluene [6,7,8]. Besides, 20 years ago, a review paper pointed out the difficulties in Rh extraction related to ageing of the Rh samples, complex speciation and slow extraction kinetics in aqueous chloride medium [9]. Among the numerous chloro-complexes of Rh(III), which range from the cationic hexaaquo-rhodate [Rh(H_2_O)_6_]^3+^, to the anionic hexachloro-rhodate [RhCl_6_]^3−^, the latter, together with the [RhCl_5_(H_2_O)]^2−^, are claimed to dominate the speciation at high HCl concentrations and are thus the focus of extraction studies [5,7].

Rh speciation is not driven solely by the concentration of the chloride ligand, but also by the ligand to metallic ion ratio, R = [Cl^−^]/[Rh(III)]. In aqueous solutions mimicking highly concentrated industrial solutions arising from ore leaching with HCl, the total ionic strength is also of great importance to the Rh speciation. Depending on R and metal concentration, the total ionic strength can, or cannot be approximated to the total HCl concentration. Obviously, ageing and kinetics are also dependent on R. All these factors may explain, at least in part, the difficulties encountered to achieve a clear understanding of Rh speciation in concentrated HCl aqueous solutions, and this renders Rh extraction a tricky story.

In this work, we first investigated Rh(III)-Cl^−^ speciation for R values ranging from ca. 310 to 5000. Over 20 UV-vis spectra were collected for various R values allowing for Principal Component Analysis (PCA) and Multivariate Curve Resolution techniques (MCR) to be applied. From this, the speciation diagram of Rh under the chemical conditions of this work could be deduced and the stoichiometry of the complexes were proposed. Then, the classical extended Debye-Hückel expression for the activity coefficient was used in order to derive the successive thermodynamic complexation constants. Application of these findings was done by comparing the distribution coefficient obtained for a phosphonium-based IL as a function of R and the speciation diagram, to finally derive the stoichiometry of the extracted species.

## 2. Results

### 2.1. Kinetics at Fixed Rh and HCl Concentrations

All the UV-vis patterns recorded from t = 1 h to at least 100 h at fixed HCl concentration appeared perfectly stable, evidencing the absence of any noticeable changes in the Rh(III) speciation in this time window (data not shown) for R values ranging from 312 to 5000 (i.e., HCl values equal or larger than 0.75 M and (Rh) = 2.4 × 10^−3^ M). By contrast, UV-vis spectra recorded at R = 208, corresponding to (HCl) = 0.5 M clearly evidenced a kinetic phenomenon which was still not finished at t = 164 h (see supplementary material, Figure S1), thus confirming the difficulty of obtaining stable Rh solutions under specific chemical conditions.

Our results are somewhat in disagreement with the kinetic study performed by Sanchez and co-workers [10], who evidenced significant UV-vis changes for R = 2060 corresponding to (Rh) = 1.94 × 10^−3^ M and (HCl) = 4 M in the time window t = 0 to t = 24 h, with no further changes above t = 24 h. Note that in another work, no effect of ageing (i.e., two weeks after preparation) was observed for Rh(III) extraction from almost similar initial concentration ((Rh) = 2.5 × 10^−3^ M) [6], while a paper cited in [9] indicated effects after seven months of ageing, a duration far above the time frame of our experiments. As a conclusion, it seems that the question of the stability of Rh aqueous solutions is still a confusing topic. Consequently, the UV-vis data at 0.5 M HCl were not be considered further.

### 2.2. Rh Aqueous Speciation

**Figure 1:**
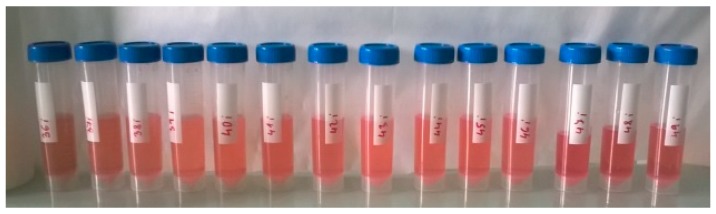
Snapshot of Rh aqueous solutions (2.4 × 10^−3^ M), ranging from (HCl) = 0.5 M on the left to (HCl) = 8 M on the right.

**Figure 2:**
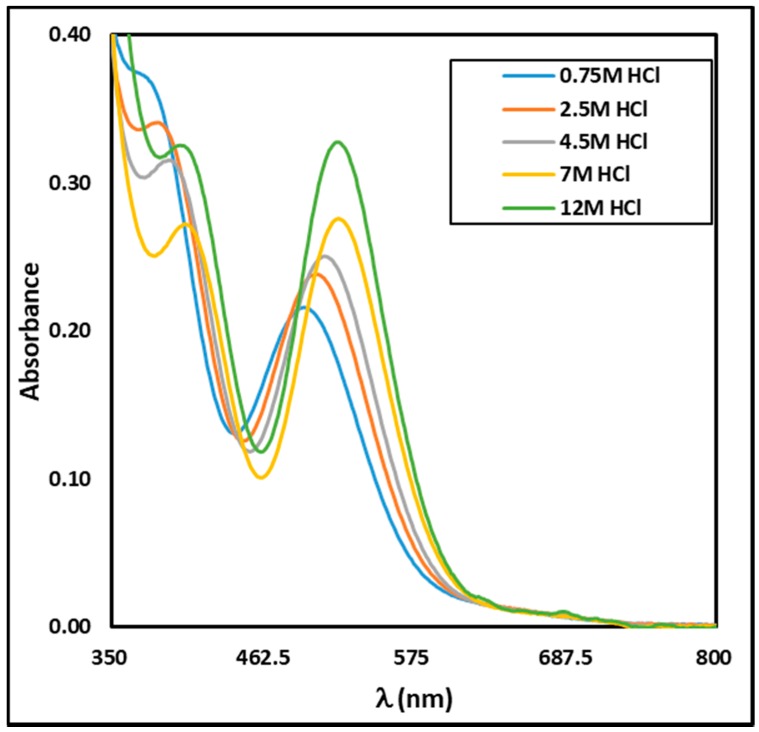
Some experimental UV-vis spectra of the Rh aqueous solutions (2.4 × 10^−3^ M)—influence of HCl concentration.

Although all prepared solutions appeared pink, their shades varied slightly with the increase in HCl concentration (Figure 1). In total, 21 spectra were obtained for HCl concentration ranging from 0.75 M to 12 M and a selection of them are displayed in Figure 2. One can notice an important shift of the characteristic peaks with the increase in HCl concentration. At 0.75 M HCl a peak is visible at 496 nm together with a shoulder at ca. 380 nm. As the HCl concentration increased, the peak shifted to the right to attain ca. 519 nm at 5.5 M HCl and did not seem to move further beyond this HCl concentration. On the other hand, the shoulder at 380 nm seemed to progressively shift towards 406 nm, becoming more pronounced. 

**Figure 3:**
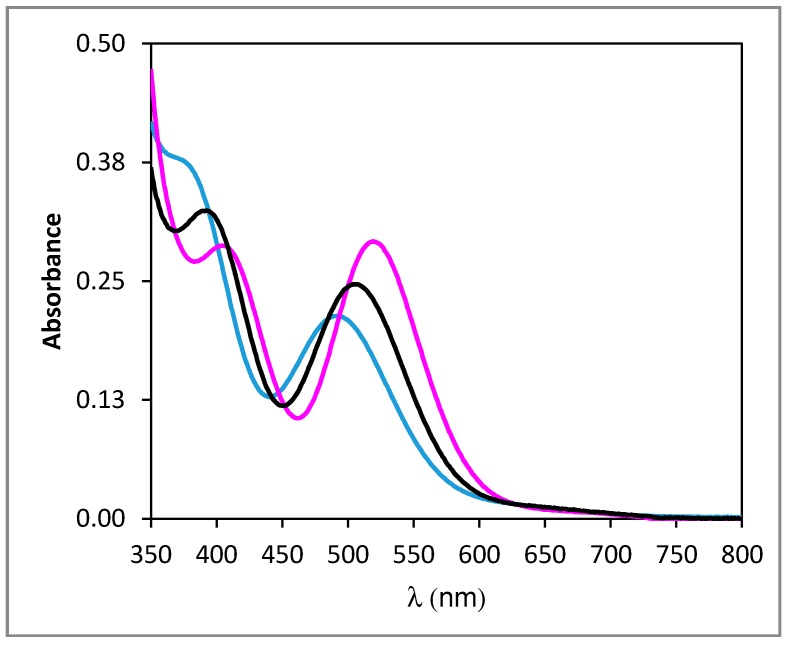
Individual UV-vis spectra of [RhCl_4_]^−^ (blue line), [RhCl_5_]^2−^ (black line) and [RhCl_6_]^3−^ (pink line) as derived from the MCR analysis of the experimental UV-vis data (see text for assignment).

**Figure 4:**
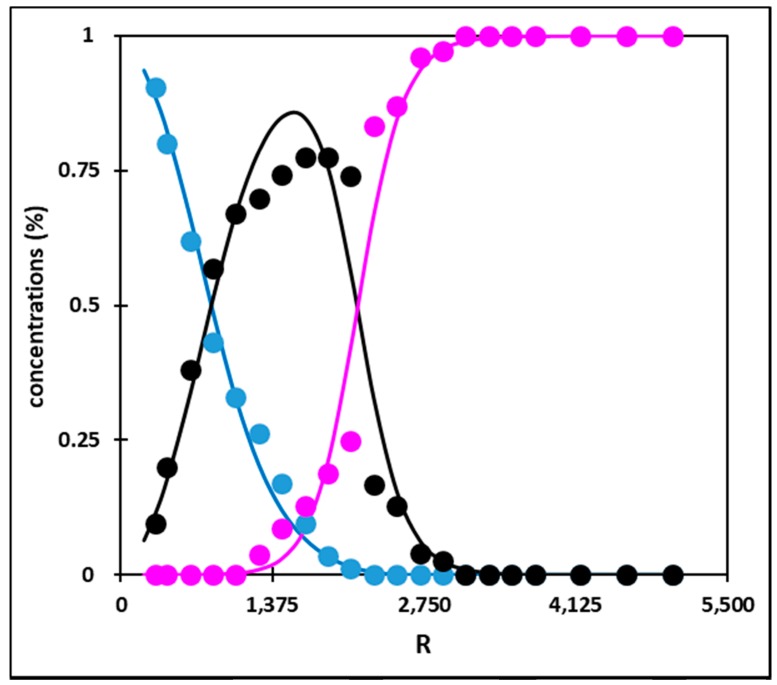
Concentration profiles of [RhCl_4_]^−^ (blue symbols), [RhCl_5_]^2−^ (black symbols) and [RhCl_6_]^3−^ (pink symbols) along the R values as derived from the MCR analysis of the experimental UV-vis data (see text for assignment). Solid lines: Fits of the data using the extended Debye-Hückel expression (see text).

A few publications have already displayed UV-vis data of Rh(III) under various chloride conditions [5,10,11,12]. However, comparing these data to ours is not possible, owing to several reasons. On an experimental point of view, comparison is difficult as the exact blank procedures do not match from one piece of work to another. Furthermore, in one paper [12], a quantitative comparison was not possible because the peak maxima were not mentioned. On a more fundamental aspect, comparison between data from different works would be possible only for similar total chloride and Rh amounts. Unfortunately, this is not the case, as Firmansyah et al. [5] presented two UV-vis spectra obtained for (Rh) = 100 mg·L^−1^ (i.e., 9.7 × 10^−4^ M) and (HCl) = 0.5 M or 5 M, respectively. Similarly, in the work of Samuels et al. [11], the closest Rh concentration to ours is equal to ca. 1.1 × 10^−3^ M. More than doubling the Rh total concentration obviously has an impact onto Rh speciation and consequently on absorbance values. The PCA analysis indicates the existence of three independent species. On this basis, the MCR analysis was then performed and led to the individual UV-vis spectra of these three species (Figure 3), together with their concentration profile (Figure 4). Table 1 gathers the local maxima of these species. As expected, the experimental UV-vis spectrum at R = 5000 matches with the fitted individual spectrum of the species present above R = 3000.

It is important to be aware that in the absence of any chemical assumption, which is the strength of such an analysis, no chemical identification in terms of stoichiometric speciation is feasible at this stage. However, a chemical assignment is possible by further examination of literature data. The data in Figure 4 are typical of successive equilibrium reactions, where the species which dominates the Rh speciation at low R values is depleted in favor of a second species, which is the major species from ca. R = 830 to R = 2000 and abruptly disappears in favor of a third species dominating Rh species above R = 2000. On the basis of Rh(III) speciation as reviewed previously [9] it can thus be reasonably assumed that the three species are [RhCl_4_]^−^, [RhCl_5_]^2−^ and [RhCl_6_]^3−^, respectively, where the H_2_O molecules completing the first coordination sphere are not mentioned for clarity. This assignment is used in Figure 3 and Figure 4 and in Table 1, which also presents the local maxima assigned in the work of Firmansyah et al. [5] for [RhCl_6_]^3−^ and [RhCl_5_]^2−^. It is important to note that [RhCl_4_]^−^ was not considered at all in this previous work, although the R range investigated started at R = 103. Interestingly, Figure 3 and Table 1 show that [RhCl_4_]^−^ and [RhCl_5_]^2−^ have rather similar absorption spectra while Figure 4 indicates that at R ≈ 625, the experimental UV-vis spectrum is composed of ca. 62% of [RhCl_4_]^−^ and 38% of [RhCl_5_]^2−^.

The two successive complexation equilibria can be written as:

(1) $${\mathrm{RhCl}}_{4}^{-}+{\mathrm{Cl}}^{-}\Leftrightarrow {\mathrm{RhCl}}_{5}^{2-}$$

(β_1_) (scheme 1)

(2) $${\mathrm{RhCl}}_{5}^{2-}+{\mathrm{Cl}}^{-}\Leftrightarrow {\mathrm{RhCl}}_{6}^{3-}$$

(β_2_) (scheme 2)

where β_1_ and β_2_ are their respective equilibrium constants. Under this frame, the association of any of the chloro-rhodate anions with H^+^ to give an acid in its neutral form was neglected, and, in particular, the existence of HRhCl_4_. Although H_2_PtCl_6_ and HAuCl_4_ are a commercially available compounds, to the best of our knowledge, HRhCl_4_ is not available. We could only find a single publication mentioning the possible existence of HRhCl_4_ but without any experimental proof or any pk_a_ value [1].

Considering the huge variation of the ionic strength, I, in the samples, ranging from 0.75 M up to 12 M, owing to HCl changes, it is unlikely that β_1_ and β_2_ would display constant values in the whole HCl investigated range. As previously noted, the Rh(III) concentration is too low to have any significant impact on the I value. Several expressions have been proposed in order to take into account the ionic strength effect onto the activity coefficients, but it is out of the scope of this work to discuss their range of validity or their effectiveness. In order to derive a tractable mathematical expression to be fitted to the data in Figure 4, a simplified version of the well-known Extended Debye-Hückel expression for the activity coefficient was used as follows:

(3) $$\log_{10}\gamma =\frac{{\mathrm{Az}}^{2}\sqrt{I}}{1+B\sqrt{I}}+CI$$

where γ is the activity coefficient of a given species, z its charge, I the ionic strength and A, B and C are fitting parameters. We chose this type of expression because it has been successfully used for UV-vis data of the system Cu(II)-LiCl-H_2_O, coupled with PCA and MCR analysis [13]. The fit of the three experimental concentration profiles all together (symbols in Figure 4) was then performed using equation 3 for the activity coefficients. The fitted curves are shown in Figure 4 (solid lines). As can be seen, the agreement between the fitted and experimental concentration profiles is very good. On this basis, the two thermodynamic complexation constants are β_1_ = 0.12 and β_2_ = 8.3 × 10^−6^.

### 2.3. Extraction

Taking into account the initial color of Rh solutions and of P_66614_Cl (transparent to slightly yellow) it was possible to visually observe the efficiency of extraction during the experiments.

**Figure 5:**
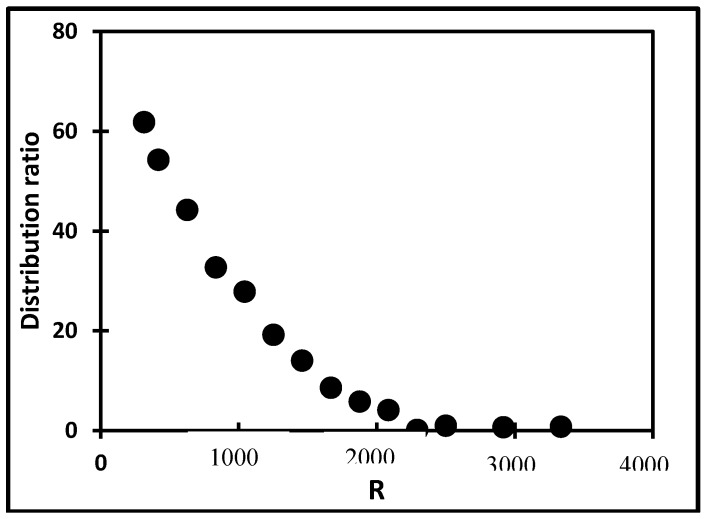
Influence of R on Rh extraction by P_66614_Cl (duplicates, A/O = 3, 24 h, room temperature, (Rh) = 2.4 × 10^−3^ M).

The results are displayed in Figure 5 and quantified in Table 2. A clear decrease in distribution coefficient from ca. 62 at R = 312 to 4 at R = 2083 can be noticed. Above this R value, D is below 1. This decreasing trend is in agreement with our previous work [4], where a limited number of experimental values were obtained. The differences between D values from our two studies are ascribed to a change in the Rh salt nature and to different ionic liquid batches. 

### 2.4. Connection between Aqueous Speciation and Extraction

The similarity between the decreasing trend of D as a function of R (Figure 5) and the decreasing behavior of [RhCl_4_]^−^ concentration profile (Figure 4) is striking. The experimental evidences of this work are (i) the high value of D while [RhCl_4_]^−^ is the main species present, (ii) the decrease in D as [Cl^−^] increases, (iii) the decrease in [RhCl_4_]^−^ amount as [Cl^−^] increases, (iv) the negligible values of D for R values above 2291, as [RhCl_6_]^3−^ represents more than 83% of the speciation and finally, (v) the rather limited extraction of [RhCl_5_]^2−^ at R = 1800, although [RhCl_5_]^2−^ is at its maximum. 

Once these evidences are stated, two entangled questions arise: What is the extraction mechanism and which is the species mainly liable to this mechanism? Based on the large D values for large [RhCl_4_]^−^ amounts, the proposed mechanism cannot cope without involving [RhCl_4_]^−^. Rh extraction can be either described by an ion exchange model or by an ion pair formation process [14]. The ion exchange model can be written as:

(4) $${\mathrm{RhCl}}_{4}^{-}+\bar{{\mathrm{Cl}}^{-}}\Leftrightarrow \bar{{\mathrm{RhCl}}_{4}^{-}}+{\mathrm{Cl}}^{-}$$

where species capped with a bar correspond to species in the IL phase. Conversely, the ion pair model is based on the fact that although hydrophobic, P_66614_Cl is nevertheless partly soluble in water, as clearly demonstrated in [5]. Therefore, the following extraction scheme can also be envisioned:

(5) $${\mathrm{RhCl}}_{4}^{-}+P_{66614}^{+}\Leftrightarrow \bar{{\mathrm{RhCl}}_{4}^{-}}+\bar{P_{66614}^{+}}$$

As previously discussed [14], these two chemical models are actually equivalent, owing to the solubility of P_6664_Cl in water.

On this basis, two phenomena are at work to explain the decrease in D as [Cl]^−^ increases. First, le Chatelier’s principle applies to equation 4. Second, the decrease in [RhCl_4_]^-^ in favor of [RhCl_5_]^2−^ and [RhCl_6_]^3−^ at higher chloride amounts unavoidably results in a decrease of D, as the extractable species [RhCl_4_]^−^ disappears. Consequently, one may envision that [RhCl_5_]^2−^ and [RhCl_6_]^3−^ could experience extraction mechanisms similar to that described in equation 4, as described below

(6) $${\mathrm{RhCl}}_{n}^{(n-3)-}+(n-3)\bar{{\mathrm{Cl}}^{-}}\Leftrightarrow \bar{{\mathrm{RhCl}}_{n}^{(n-3)-}}+(n-{3)\mathrm{Cl}}^{-}$$

On this basis, and considering the decrease in D as the speciation of Rh(III) turns to higher chlorocomplexes, the conditional extraction constants corresponding to [RhCl_5_]^2−^ and [RhCl_6_]^3−^ extraction are much lower than that for [RhCl_4_]^−^. Thus, these species do not contribute significantly to the extraction mechanism.

The extraction of Rh(III) from hydrochloric aqueous solutions towards a newly synthesized ionic liquid, P_88812_Cl (trioctyl(dodecyl)phosphonium chloride), either pure or diluted in toluene has been investigated in details [5]. In this previous work, the Rh total concentration in the aqueous phase prior to extraction is equal to 100 mg·L^−1^, i.e., 9.7 × 10^−4^ M. According to the authors, starting from R = 103, Rh extraction increases until R equals 515 and then decreases sharply up to R = 5155, the maximum R value that was investigated. The authors ascribed these changes to a change in the aqueous speciation of Rh(III), indicating that [RhCl_6_]^3−^ dominates the speciation for R > 3092 and is not favorably extracted, while [RhCl_5_]^2−^ is the extracted species, owing to its higher charge density. The increase in D at low R values would thus correlate with an increase in [RhCl_5_]^2−^ percentage, and [RhCl_5_]^2−^ should be maximum at R = 515. This is not in line with our speciation diagram as illustrated in Figure 4. Kinetics effects at low R values and the omission of [RhCl_4_]^−^ as a possible extractable species may explain these discrepancies.

**Figure 6:**
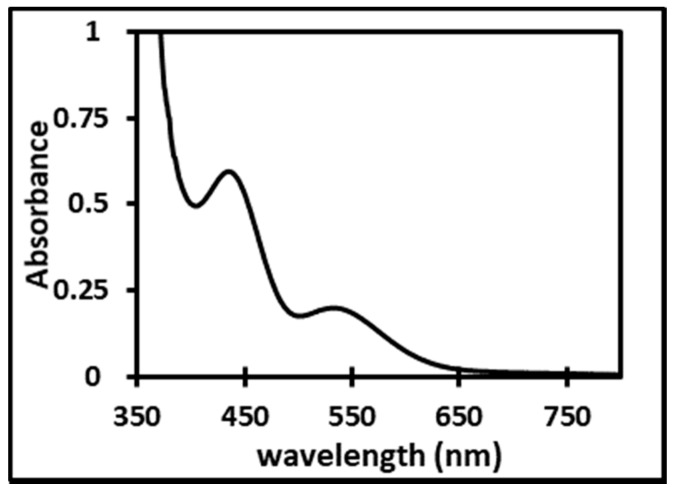
Absorption spectrum of the Rh species extracted in the IL phase.

Figure 6 displays the absorption spectrum of the Rh complex present in the IL phase just after extraction is completed.

The two peaks (λ_1_ = 435 nm and λ_2_ = 534 nm) are separated by ca. 110 nm, just as for the three aqueous species (see Table 1) and a clear shift of the whole spectrum is observed as compared to the species in water. This is expected as the Rh complex is not in the same solvent.

## 3. Materials and Methods 

### 3.1. Chemicals and Stock Solutions

All chemicals were used as received without any further purification, and all experiments were carried-out at room temperature. Ammonium hexachlororhodate(III) hydrate ((NH_4_)_3_RhCl_6_·*x*H_2_O) salt was purchased from Alfa Aesar (Karlsruhe, Germany). Concentrated HCl (37% by weight) was supplied by Sigma Aldrich (Saint Quentin Fallavier, France). The ionic liquid trihexyltetradecylphosphonium chloride, [P_66614_][Cl] (Cyphos 101) was kindly provided by Cytec Industries (Pont de Claix, France). All aqueous samples were prepared with ultra-pure water (Merck Millipore, system Milli-Q Integral 5, Molsheim, France). A rhodium initial stock solution (1 g Rh L^−1^) in 1 M HCl was prepared from the ammonium hexachlororhodate salt. This solution was further diluted with water or concentrated HCl acid in order to obtain the expected HCl concentrations.

### 3.2. UV-vis Measurements of the Aqueous Phase

The UV-vis spectra of Rh solutions were recorded in the range of 350–800 nm, with a reference sample composed of ultra-pure water. First, at given Rh and HCl concentrations, a possible kinetics was investigated, and, second, the change in Rh speciation as a function of HCl concentration was looked for. Rh kinetics was followed from the preparation moment up to 100 h for a constant Rh concentration of 2.4 × 10^−3^ M (i.e., 250 mg·L^−1^) and for HCl concentrations ranging from 0.5 M to 12 M. Consequently, R values covered the range between 300 to 5000, and the ionic strength could safely be calculated based on the HCl concentration only. Rh speciation was examined at the same constant Rh concentration of 2.4 × 10^−3^ M and for the same range of HCl concentrations at t = 24 h after the preparation moment. All the samples were kept in dark between their preparation and their analyses.

### 3.3. Extraction Experiments

The extraction experiments were carried out by varying the initial HCl concentration from 0.75 to 8 M while keeping the rhodium concentration constant at 2.4 × 10^−3^ M. The aqueous to organic volume ratio A/O were fixed to approximately 3. Typically, 1.2 g of IL was put into contact with 4 mL of rhodium solution in a PP test tube and agitated for 24 h at room temperature. The start of the extraction experiments have always been performed at t = 24 h after the preparation time, so phase separation occurred at t = 48 h after the aqueous Rh solution preparation. Then, the phases were separated by centrifugation at 4000 rpm for 20 min. All extraction experiments were performed in duplicates. No volume changes were observed during the extraction process. The final rhodium content within aqueous phases was analyzed by atomic absorption spectroscopy (AAS) and compared to the initial Rh content. Finally, the extraction efficiency (E%) together with distribution coefficient (D) were calculated as follows:

(7) $$D=\frac{(C_{i}-C_{f})*V_{aq}}{C_{f}*V_{IL}}$$

(8) $$E\%=(1-\frac{C_{f}}{C_{i}})*100$$

where C_i_ and C_f_ are respectively the initial and the final metal concentrations in the aqueous phase and *V_aq_* and *V_IL_* are the volumes of aqueous and ionic liquid phases, respectively. Experimental uncertainties on D and E% are 2% and 5%, respectively. 

For an IL phase loaded with the Rh complex ((Rh) = 2.4 × 10^−3^ M and (HCl) = 0.5 M), a UV-vis spectrum was recorded in the range 350–800 nm (blank sample: IL saturated with (HCl) = 0.5 M).

### 3.4. UV-Vis Spectra Analysis and Data Fitting

The 21 UV-vis spectra recorded at t = 24 h for R = 312 to 5000 ([Rh] = 2.4 × 10^−3^ M and [HCl] varying from 0.75 M to 12 M) were analyzed using Matlab dedicated routines for Principal Component Analysis and Multivariate Curve Resolution. From this, the UV-vis spectra of the independent aqueous Rh species were obtained in the range 350–800 nm, together with the percentage distribution of these species in the 21 UV-vis spectra. In the following, this percentage distribution will be referred to as the experimental concentration profile of species X.

Then, assuming a chemical scheme (see Section 2.2), a theoretical expression relating the calculated concentration profile of all the independent species to the chemical conditions (HCl and Rh total concentrations), was derived. This mathematical expression was fitted to the experimental concentration profiles in order to derive the value of the equilibrium reaction constants of the chemical scheme of interest. This fitting procedure was performed with the use of a dedicated Fortran subroutine, and taking advantage of the CERN fitting facilities. Minimization was performed under a classical Marquardt least-square procedure, using the following *χ*^2^ definition:

(9) $$\chi^{2}=\sqrt{\sum_{X}^{}\sum_{j=1,\;21}{\left(\mathrm{Exp}(X,j\right)-\mathrm{Cal}(X,j))}^{2}}/N$$

where N is the total number of data to be fitted, X is the independent species of concern and Exp(X,j) and Cal(X,j) are the experimental and the calculated contribution of species X to the jth UV-vis spectrum, respectively.

## 4. Conclusions

Under conditions that prevent any kinetic effect, the speciation of Rh(III) in H_2_O/HCl medium was investigated up to very high chloride-metal ratios by collecting UV-vis spectra. These data were liable to a PCA and a MCR analysis, allowing the speciation diagram to be drawn, thermodynamic constants to be derived and the stoichiometry of the various species to be given. Comparison with the Rh(III) extraction profile towards a pure phosphonium-based IL evidences the interest of such speciation studies in order to decipher the extraction mechanism.

Some studies have been using extraction experiments in order to decipher speciation of metallic ions in the aqueous phase, in particular when handling of such elements is not easy (for radioactive reasons, for example) [15]. Upon changing the aqueous chemical conditions, the metallic ion is, or is not, extracted towards a molecular solvent, which in turn allows for the identification of the extracted species, which has to be neutral in the molecular solvent, thus giving information on the aqueous speciation, and on ionic strength effects, as the two final goals. This work is the mirror experiment of these studies: By understanding the aqueous speciation, the extraction mechanism towards the ionic liquid phase can be understood. 

Finally, the interest of the PCA and MCR analysis was evidenced, and this method will soon be used in order to confirm the stoichiometry of the Rh extracted species as shown in Figure 6. 

## Figures and Tables

**Table 1:** Values of the local absorption maxima for the three Rh-chloro complexes as derived from this work. Values from [5] in parenthesis.

Species	λ_1_ (nm)	λ_2_ (nm)
[RhCl_4_]^−^	Shoulder at ≈ 381 (none)	492 (none)
[RhCl_5_]^2−^	392 (402.5)	507 (505)
[RhCl_6_]^3−^	404 (411.5)	519 (519.5)

**Table 2:** Influence of HCl concentration on Rh extraction efficiency. Corresponding R values in parenthesis.

HCl Concentration (M) (R)	Extraction Efficiency (%)	Distribution Coefficient (−)
0.75 (312)	94.87	61.83
1.00 (417)	94.56	54.30
1.50 (625)	93.14	44.26
2.00 (833)	91.29	32.75
2.50 (1042)	89.87	27.86
3.00 (1250)	86.63	19.22
3.50 (1458)	81.81	14.04
4.00 (1667)	74.68	8.62
4.50 (1875)	65.93	5.85
5.00 (2083)	55.04	4.14
5.50 (2292)	6.25	0.16
6.00 (2500)	22.15	0.99
7.00 (2917)	19.14	0.69
8.00 (3333)	19.29	0.81

## Supplementary Materials

The following are available online, Figure S1: UV-vis absorption of Rh(III) (2.4 × 10^−3^ M) in H_2_O/HCl 0.5 M as a function of time after preparation.
