# Supplementary material for: Rh(III) Aqueous Speciation with Chloride as a Driver for Its Extraction by Phosphonium Based Ionic Liquids
Source: Molecules. 2019 Apr 9;24(7):1391. doi: 10.3390/molecules24071391 (PMC6480558; doi:10.3390/molecules24071391)
Supplement: Supplementary file 1 [file molecules-24-01391-s001.pdf]

Supplementary materials:

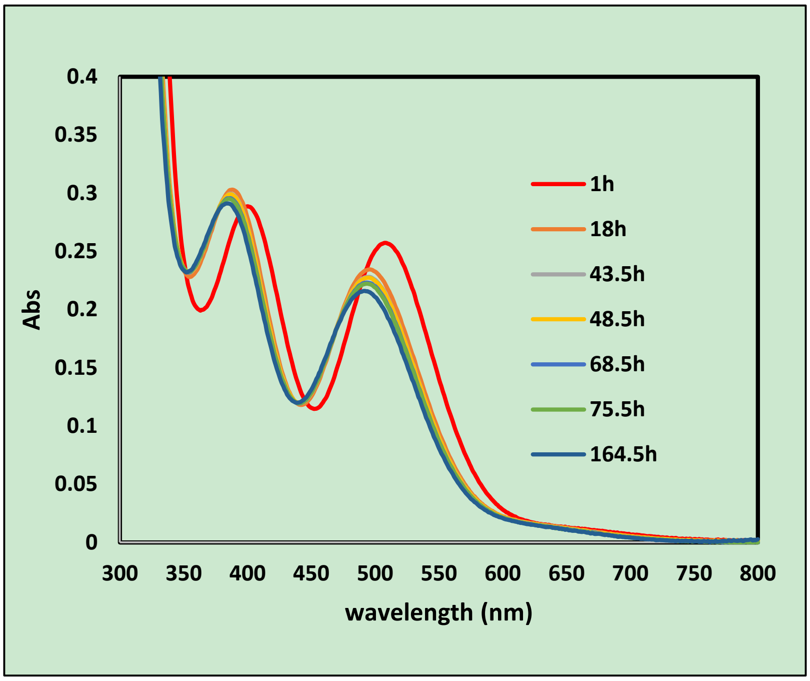

Fig. S1: UV-vis absorption of Rh(III) ($2.4x10^{-3}$ M) in H_2O/HCl 0.5 M as a function of time after preparation.
